# CD4^+^ T-cell-dependent differentiation of CD23^+^ follicular B cells contributes to the pulmonary pathology in a primary Sjögren’s syndrome mouse model

**DOI:** 10.3389/fimmu.2023.1217492

**Published:** 2023-07-05

**Authors:** Mami Sato-Fukuba, Rieko Arakaki, Aya Ushio, Kunihiro Otsuka, Ruka Nagao, Shigefumi Matsuzawa, Hiroaki Tawara, Takaaki Tsunematsu, Naozumi Ishimaru

**Affiliations:** ^1^ Department of Oral Molecular Pathology, Tokushima University Graduate School of Biomedical Sciences, Tokushima, Japan; ^2^ Department of Oral Medicine, Tokushima University Hospital, Tokushima, Japan

**Keywords:** primary Sjögren’s syndrome, pulmonary lesion, CD23, follicular B cell, bronchus-associated lymphoid tissue, CD4^+^ T cell

## Abstract

**Introduction:**

Primary Sjögren’s syndrome (pSS) is a systemic autoimmune disease that affects the function of exocrine glands, such as the lacrimal and the salivary glands. Extraglandular lesions and malignant lymphoma also occur during the progressive stage of pSS. We have, herein, focused on the pulmonary lesions of pSS and have aimed clarifying their pathophysiological mechanism by comparing the glandular with the extraglandular lesions observed in a mouse model of pSS.

**Results:**

The histopathological analysis of lung tissues obtained from NFS/*sld* mice that have undergone neonatal thymectomy was performed. Moreover, *in vivo* and *in vitro* experiments were conducted along with immunological analyses in order to characterize the unique phenotypes of the pulmonary lesions identified in these pSS model mice. Inflammatory lesions with a bronchus-associated lymphoid tissue-like structure were identified in the lungs of pSS model mice. In addition, relative to salivary gland lesions, pulmonary lesions showed increased CD23^+^ follicular B (FB) cells. *In vitro* and pulmonary B cells were more readily driven to CD23^+^ FB cell phenotype than salivary gland B cells in pSS model mice. Furthermore, the CD23^+^ FB cell differentiation was found to be enhanced in a CD4^+^ T-cell-dependent manner under a Th2-type condition in the lungs of herein examined pSS model mice.

**Discussion:**

A Th2-type response in the pSS lung may promote the progression of autoimmune lesions through an enhanced abnormal differentiation of B cells.

## Introduction

Primary Sjögren’s syndrome (pSS) is a systemic autoimmune disease that affects the exocrine glands, such as the lacrimal and the salivary glands (1–3). In addition, various extraglandular lesions may occur in the lung, the kidney, the joints, or the lymph nodes in many patients with this syndrome (4, 5). Systemic symptoms in pSS patients are well known and include arthralgia, fatigue, general discomfort, and cardiovascular events (6, 7). Furthermore, non-Hodgkin’s lymphoma, including mucosa-associated lymphoid tissue (MALT) lymphoma, develops in some patients with pSS (8–12). However, it is still unclear how a wide spectrum of target organs can be involved in the pathogenesis of pSS.

The development of pulmonary lesions in pSS is sometimes severe (13, 14). These lesions are expressed as airway disease and interstitial lung disease (15, 16). Lymphocytic interstitial pneumonia (LIP) is one of the pulmonary lesions occurring in patients with pSS, and the 5-year mortality of LIP is estimated to be between 33% and 50% (17). Moreover, pulmonary lesions with inflammation in several secondary or pSS model mice have been reported (18–20). Although the autoimmune pathology has been identified in exocrine glands, such as the salivary glands and the lacrimal glands, its precise molecular or cellular mechanisms are unclear in the case of the pulmonary lesions in pSS. T cells predominately infiltrate the salivary glands at the early stage of pSS (21). According to the disease activity, B cells or plasma cells prevail in the chronic lesions of the salivary glands (21, 22). In addition, various immune cells, such as macrophages, dendritic cells, and lymphoid innate cells, are known to contribute to the formation of the inflammatory lesions and the pathogenesis of pSS (23–25). However, the immunopathological differences between the glandular and the extraglandular lesions in pSS have not yet been fully understood. The undertaking of a precise analysis through the use of a mouse model that exhibits both glandular and extraglandular lesions would facilitate our understanding of the pathogenesis of this systemic autoimmune disease.

In the present study, the autoimmune pathology of the pulmonary lesions was investigated by using a mouse model of pSS in which autoimmune lesions are observed both in the salivary glands and in the lungs. The findings of this study may allow us to gain insight into a novel pathogenic mechanism of autoimmunity and may also facilitate the potential establishment of new treatments for autoimmune diseases such as the pSS.

## Materials and methods

### Mice

NFS/*sld* mice were obtained from the Central Institute for Experimental Animals (Kawasaki, Japan). Neonatal thymectomy (Tx) was performed to female NFS/*sld* mice on day 3 after birth in order to establish the pSS model. At 4 weeks after the Tx surgery, peripheral blood mononuclear cells from the tail vein of these mice were analyzed by using flow cytometry. The completeness of the Tx surgery was confirmed by evaluating the proportion of CD90.2^+^ T cells (<7%). The control mice used in this study were sham (non)-thymectomized female NFS/*sld* mice. Female MRL/MpJ-Fas*^lpr/lpr^* (MRL/*lpr*) and *aly/aly* mice were obtained from Japan SLC Inc. (Hamamatsu, Japan). The total number of mice used in this study is 249 mice. Mice were housed at 21°C–23°C on a 12-h light/dark cycle in our facility under specific pathogen-free condition. The present study was designed and undertaken by following the “Fundamental Guidelines for Proper Conduct of Animal Experiments and Related Activities in Academic Research Institutions” under the jurisdiction of the Ministry of Education, Culture, Sports, Science, and Technology of Japan. The protocol was approved by the Committee on Animal Experiments of Tokushima University (permit number T2021-48). Experiments were performed under general anesthesia, and all efforts were made so as to minimize the suffering of the mice involved. Randomization was performed to allocate experimental units to all mice used in each experiment. In order to establish the pulmonary fibrosis model, the mice received a transbronchial instillation of 1.675 mg/kg (body weight) bleomycin (Nippon Kayaku Co.,Ltd.). Three weeks after the bleomycin administration, the mice were euthanized for the undertaking of the following analyses.

### Histological analyses

Lung tissues were fixed with 10% phosphate-buffered formalin (pH 7.2) and were prepared for histological examination. Sections were stained with hematoxylin and eosin (HE) or Masson’s trichrome staining. The focus score in the lung tissues was determined by counting as foci the visible aggregations containing 50 or more tightly aggregated lymphocytes. In addition, the number of foci infiltrated in the entire tissue section was counted per mm^2^. The area of foci was measured using NIS Elements D (Nikon Corporation). For the semiquantitative evaluation of fibrosis in the lungs, histological analysis was performed by using the Ashcroft score (26). In brief, the degree of fibrosis was scored from 0 to 5 as follows: 0 = normal; 1 = presence of inflammation and fibrosis involving <5% of the lung parenchyma; 2 = presence of inflammation and fibrosis involving <25% of the lung parenchyma; 3 = lesions involving 25%–50% of the lung parenchyma, accompanied by moderate thickening of the bronchial wall without obvious damage to the lung architecture, or small fibrous masses; 4 = lesions involving >50% of the lung, parenchyma, accompanied by definite damage to the lung structure and formation of fibrous bands or small fibrous masses; 5 = lesions involving >50% of the lung parenchyma accompanied by severe distortion of the lung structure and a fibrous obliteration of fields. All evaluations were performed in a blind manner by three pathologists.

### Immunofluorescence analysis

Lung tissue sections were deparaffinized in xylenes and were rehydrated by passage through serial dilutions of ethanol in distilled water. Heat-induced antigen retrieval was performed in an antigen retrieval solution (ImmuoActive; Matsunami Glass Ind., Ltd., Kishiwada, Japan) with the use of a microwave, twice, for 5 min. The sections were incubated with Blocking One Histo (Nacalai Tesque) in order to block non-specific reactions. Anti-mouse B220 (eBioscience), anti-mouse CD3 (Cell Signaling Technology) monoclonal antibodies (mAbs), and anti-mouse CD23 (Boster Biological Technology) polyclonal Ab were applied to the sections overnight, at 4°C. After washing with phosphate-buffered saline (PBS), the sections were incubated with an Alexa Fluor 488-conjugated anti-rat IgG (Invitrogen) or an Alexa Fluor 568-conjugated anti-rabbit IgG (Invitrogen) Ab. After washing thrice with PBS, the nuclear DNA was stained with 4′,6-diamidino-2-phenylindole (DAPI; Invitrogen, Waltham, MA), and the sections were observed under an optical microscope (ZEISS Axio Observer 7; Carl Zeiss) and were analyzed through the use of ZEN3.2 blue edition (Carl Zeiss).

### Immunohistochemistry

Deparaffinized sections from lung tissues were used. Heat-induced antigen retrieval was performed in ImmunoActive, and the sections were incubated with Blocking One Histo. Subsequently, the sections were incubated with anti-mouse CD3 (Cell Signaling Technology), CD19 (Cell Signaling Technology, Danvers, MA), F4/80 (Cell Signaling Technology), CD11b (Cell Signaling Technology), or CD23 (Boster Biological Technology) Abs overnight, at 4°C. After washing with PBS, the sections were incubated with a horseradish peroxidase (HRP)-conjugated secondary Ab (Cell Signaling Technology). HRP reacted with the 3,3′-diaminobenzidine (DAB) substrate through the use of SignalStain DAB Substrate Kit (Cell Signaling Technology), and the sections were then counterstained with hematoxylin.

### Cell isolation

Under anesthesia, PBS was perfused into the left ventricle of each mouse in order to remove hematopoietic cells among the blood. For the isolation of lymphocytes from the lung and the salivary gland tissues, the tissues were minced into 1–3-mm pieces and were digested with collagenase (1 mg/ml; FUJIFILM Wako Pure Chemical Corp., Osaka, Japan), hyaluronidase (1 mg/ml; Sigma-Aldrich Co., St. Louis, MO), and DNase (10 μg/ml; Roche Diagnostics K.K., Tokyo, Japan) in Dulbecco’s modified Eagle’s medium (DMEM) containing 10% fetal bovine serum (FBS), at 37°C, for 30 min, by using gentleMACS Dissociators (Miltenyi Biotec, Bergish Gladbach, Germany). The spleen was homogenized in DMEM containing 2% FBS through the use of gentleMACS Dissociators (Miltenyi Biotec). By using 0.83% ammonium chloride, the red blood cells were removed from the tissues. In addition, both the viability and the number of the isolated cells were evaluated by a Luna II cell counter (Logos Biosystems, Anyang, South Korea) using trypan blue staining. Subsequently, the proportions of the suspended cells were analyzed by a flow cytometer. The absolute numbers of immune cells were calculated by using the data pertaining to their total cell number and their proportions. As for the lungs and the salivary glands, we used their lateral lobes in order to determine the cell numbers and the proportions of the immune cells in them. In the case of splenocytes, the whole spleen of each mouse was used in order to determine their numbers and their proportions.

### Flow cytometric analysis

Isolated lymphocytes were stained using Abs against fluorescein isothiocyanate (FITC)-conjugated anti-mouse CD21 (BD Biosciences, 7G6) CD4 (BioLegend, RM4-5), PE-conjugated anti-mouse CD23 (BioLegend, B3B4), CD19 (BioLegend, 6D5), PD-1 (BioLegend, San Diego, CA, 29F.1A12), CD45.2 (BioLegend, 104), PE-Cy7-conjugated anti-mouse CD45.2 (BioLegend, 104), CD4 (BioLegend, GK1.5), CXCR5 (Biolegend, L138D7), APC-conjugated anti-mouse CD3 (BioLegend, 17A2), CD19 (Biolegend, 6D5), Alexa flour 700-conjugated anti-mouse CD45.2 (BioLegend, 104), APC-Cy7-conjugated anti-mouse CD19 (BioLegend, 6D5), CD3 (BioLegend, 145-2C11), Brilliant Violet 510-conjugated CD4 (Biolegend, GK1.5), and Pacific Blue-conjugated CD45 (Biolegend, S18009F). A CytoFLEX S flow cytometer (Beckman Coulter, Brea, CA) was used to identify the cell populations according to expression profile. Viable cells were checked by gating on side scatter (SSC)/forward scatter (FSC), FSC-H/FSC-A, and 7-aminoactinomycin D (7AAD) staining solution (Invitrogen). Data were analyzed by using the FlowJo FACS Analysis software (BD Biosciences).

### Quantitative reverse transcription–polymerase chain reaction

Total RNA from the spleen, the lungs, and the salivary glands was extracted with RNAiso Plus (TaKaRa Bio Inc., Kusatsu, Japan) according to the manufacturer’s instructions. Total RNA was then reverse-transcribed into cDNA using the PrimeScript RT Master Mix (TaKaRa Bio Inc., Cat. No. RR037A). The transcriptions of target genes and β-actin from the tissues were generated with a Light Cycler 96 System (Roche) using TB Green Premix Ex Taq II (TaKaRa Bio Inc., Cat. No. RR820) and the following primers: *Gata3*, forward, 5′-CCTACCGGGTTCGGATGTAA-3′ and reverse, 5′-CACACACTCCCTGCCTTCTGT-3′; *Tbx21*, forward, 5′-CCTGTTGTGGTCCAAGTTCAAC-3′ and reverse, 5′-CACAAACATCCTGTAATGGCTTGT-3′; *Il4*, forward, 5′-TCTCATGGAGCTGCAGAGACTCT-3′ and reverse, 5′-TCCAGGAAGTCTTTCAGTGATGTG-3′; *Il13*, forward, 5′-AGACCAGACTCCCCTGTGCA-3′ and reverse, 5′-TGGGTCCTGTAGATGGCATTG-3′; *Il10*, forward, 5′-GCTCTTACTGACTGGCATGAG-3′ and reverse, 5′-CGCAGCTCTAGGAGCATGTG-3′; *Ifng*, forward, 5′-AGCGGCTGACTGAACTCAGATTGTAG-3′ and reverse, 5′-GTCACAGTTTTCAGCTGTATAGGG-3′; *Il6*, forward, 5′-TCCTTCCTACCCCAATTTCC-3′ and reverse, 5′-GCCACTCCTTCTGTGACTC-3′; *Ccl4*, forward, 5′-TGTCTGCCCTCTCTCTCCTCT-3′ and reverse, 5′-AGCAAGGACGCTTCTCAGTGA-3′; *Ccl6*, forward, 5′-TTATCCTTGTGGCTGTCCTTG-3′ and reverse, 5′-CACGGGATCTGTGTGGCATA-3′; *Ccl8*, forward, 5′-ACGCTAGCCTTCACTCCAAAA-3′ and reverse, 5′-TTCCAGCTTTGGCTGTCTCTT-3′; *Ccl12*, forward, 5′-TCGAAGTCTTTGACCTCAACA-3′ and reverse, 5′-GGGAACTTCAGGGGGAAATA-3′; *Ccl19*, forward, 5′-AACAAAGGCAACAGCACCA-3′ and reverse, 5′-CACACTCACATCGACTCTCTA-3′; *Ccl22*, forward, 5′-TCATGGCTACCCTGCGTGTC-3′, and reverse, 5′-CCTTCACTAAACGTGGCAGAG-3′; *Ccl28*, forward, 5′-GCTGTGTGTGTGGCTTTTCAA-3′ and reverse, 5′-TACCTCTGAGGCTCTGATCCA-3′; *Cxcl1*, forward, 5′-CTGGGATTCACCTCAAGAACATC-3′ and reverse, 5′-CAGGGTCAAGGCAAGCCTC-3′; *Cxcl2*, forward, 5′-ACCACCAGGCTACAGCGGCT-3′ and reverse, 5′-TCCTGGGGGCCTCACACTCA-3; *Cxcl12*, forward, 5′-CTTCATCCCCATTCTCCTCA-3′ and reverse 5′-GACTCTGCTCTGGTGGAAGG-3′; *Cxcl13*, forward, 5′-CATCATGAGGTGGTGCAAAG-3′ and reverse, 5′-GGGTCACAGTGCAAAGGAAT-3′; *Ccr1*, forward, 5′-ATCCTCAAAGGCCCAGAAACA-3′ and reverse, 5′- TCCTTTGCTGAGGAACTGGTC-3′; *Ccr2*, forward, 5′-CCATGCAAGTTCAGCTGCCT-3′ and reverse, 5′-TGCCGTGGATGAACTGAGG-3′; *Ccr3*, forward, 5′-TTGATCCTCATAAAGTACAGGAAGC-3′ and reverse, 5′-CAATGCTGCCAGTCCTGCAA-3′; *Ccr4*, forward, 5′-GGCTACTACGCCGCCGAC-3′ and reverse, 5′-TACCAAAACAGCATGATGCC-3′; *Ccr5*, forward, 5′-AATTCTTTGGACTGAATAACTGCA-3′ and reverse, 5′-TCAGGATTGTCTTGCTGGAA-3′; *Ccr7*, forward, 5′-ATGCTGGCTATGAGTTTC-3′ and reverse, 5′-GCTGCTATTGGTGATGTT-3′; *Cxcr5*, forward, 5′-ACTCCTTACCACAGTGCAC-3′ and reverse, 5′-GGAAACGGGAGGTGAACCA-3′; *Tgfb1*, forward, 5′-GACCGCAACAACGCCATCTAT-3′ and reverse, 5′-GGCGTATCAGTGGGGGTCAG-3′; *Il33*, forward, 5′-ATTTCCCCGGCAAAGTTCAG-3′ and reverse, 5′- AACGGAGTCTCATGCAGTAGA-3′; *Col1a2*, forward, 5′-CCAAGGGTAACAGTGGTGAA-3′ and reverse, 5′-CCTCGTTTTCCTTCTTCTCCG-3′; *Col3a1*, forward, 5′-AACGGAGCTCCTGGCCCCAT-3′ and reverse, 5′-CCATCACTGCCCCGAGCACC-3′; *Col4a1*, forward, 5′-ATGCCCTTTCTCTTCTGCAA-3′ and reverse, 5′-GAAGGAATAGCCGATCCACA-3′; and *β-actin*, forward, 5′-GACGGCCAGGTCATCACTAT-3′, and reverse 5′-CTTCTGCATCCTGTCAGCAA-3′. Relative mRNA expression of each transcript was normalized against *β-actin* mRNA.

### Administration of the mAbs

An anti-mouse CCL6 mAb (5 μg/mouse; R&D Systems) was administered every 2 days *via* intraperitoneal injections to pSS model mice from the sixth until the eight weeks of their lives. Moreover, an anti-mouse CXCL13 mAb (10 μg/mouse; R&D Systems) was administered twice a week *via* intraperitoneal injections to pSS model mice from sixth until the eight weeks of their lives. Finally, an Ultra-LEAF purified anti-mouse CD4 mAb (100 μg/mouse; BioLegend) was intraperitoneally injected twice a week into pSS model mice from the sixth until the eighth weeks of their lives. In addition, CD4 mAb was administered using the same protocol from the fourth until the sixth weeks of their lives. As a control, the Ultra-LEAF purified rat IgG2b κ isotype control Ab (5 μg/or 50 or 100 μg/mouse; BioLegend) and the Ultra-LEAF purified rat IgG2a κ isotype control Ab (10 μg/mouse; BioLegend) were administered into pSS model mice as the same regimens.

### *In vitro* culturing of CD19^+^ B cells with recombinant IL-4

Dead cells among the lymphocytes isolated from lung tissues were removed by using the Dead Cell Removal Kit (Miltenyi Biotec) for *in vitro* differentiation into CD23^+^ B cells. CD19^+^ B cells were obtained through positive selection by using the Dynabeads (Invitrogen) and an anti-CD19 mAb (eBioscience). CD19^+^ B cells were dispensed in triplicates (3.5 × 10^5^ cells/well) from 8-week-old three control mice or pSS model mice using 48-well culture plate (Corning Incorporated). B cells were cultured for 1 week in Roswell Park Memorial Institute (RPMI) 1640 containing 10% FBS with an anti-CD40 mAb (5 µg/ml; BioLegend, HM40-3) and recombinant IL-4 (100 ng/ml; BioLegend) as described in a previous report (27).

### Statistical analysis

Differences between individual groups were determined by using a Student’s *t*-test, or one or two-way analysis of variance, where *p*-values of <0.05 were considered as statistically significant. GraphPad Prism 8 software was used for all data analysis. Data are presented as mean ± standard deviation (SD).

## Results

### Pulmonary lesions in the pSS model mice

**Figure 1:**
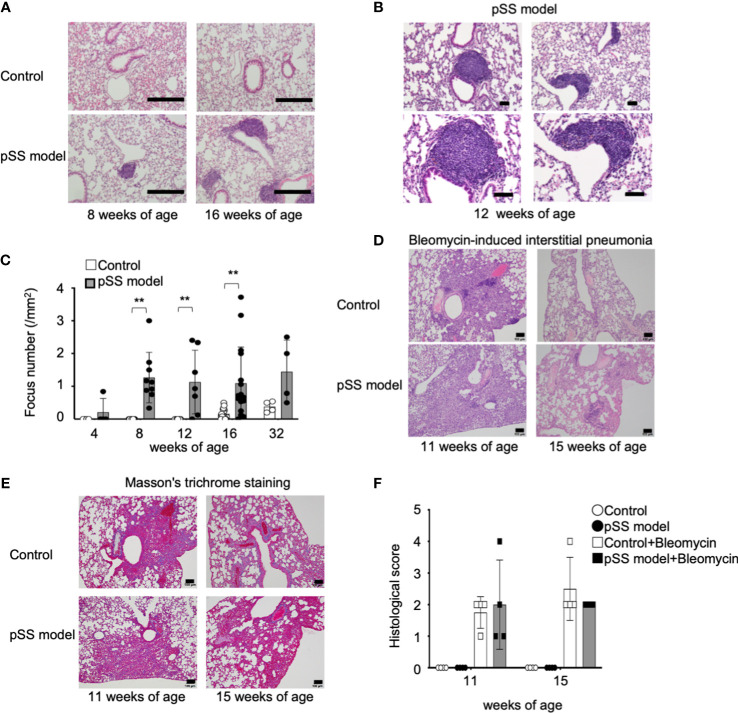
Pulmonary lesions in the pSS model mice. **(A)** Histology of the inflammatory lesions identified in the lungs of pSS model mice. Representative images depict HE-stained lung tissue sections obtained from female control and pSS model mice at their 8 and 16 weeks of age. Scale bar: 200 μm. **(B)** BALT-like structure in the pulmonary lesions of pSS model mice. Representative images of HE-stained sections obtained from 12-week-old pSS model mice. Scale bar: 50 µm. **(C)** The number of foci in the lung tissues was counted by using HE-stained sections of control and pSS model mice between 4 and 32 weeks of age. Data are presented as mean ± SD of 4−16 mice per group. **p < 0.01. **(D)** Histology of bleomycin-induced interstitial pneumonia (BIIP) in the control and the pSS model mice. Bleomycin was intratracheally administered in control and pSS model mice at 8 or 12 weeks of age. Histopathological analysis was performed 3 weeks later by using HE-stained sections of the lung tissues. Representative images of lung sections from 11- and 15-week-old mice. Scale bar: 100 µm. **(E)** Fibrotic change of BIIP in control and pSS model mice. Representative images of lung sections from 11- and 15-week-old mice. Scale bar: 100 µm. **(F)** BIIP was evaluated through the histological scoring of HE-stained lung tissue sections. Data are presented as mean ± SD of four mice per group.

We have established a mouse model of pSS by using NFS/*sld* mice treated with neonatal Tx, and the autoimmune lesions in the salivary and the lacrimal glands of these mice can be observed as early as the mice reach 6 weeks of age (28–31). The pathological analysis of the lung tissues in these pSS model mice was performed from the 4th to the 32nd week of their lives. When the mice became 8 weeks of age, a focal lymphocytic infiltration consisting of mononuclear lymphocytes was identified in the pSS model mice, while no inflammatory lesions were observed in the control mice (**Figure 1A**). Focal nodular infiltration was observed around the blood vessels and peribronchially in the pulmonary interstitium in the pSS model mice (**Figure 1B**). The pulmonary lesions in the pSS model mice resembled those of patients with pSS and those observed in bronchus-associated lymphoid tissue (BALT) forming during chronic inflammation in the lung (16, 32, 33). Interestingly, the BALT-like structure observed in these pSS model mice has not been confirmed in the pulmonary lesions of the other SS model mice, such as the MRL/*lpr* or the *alymphoplasia* (*aly)/aly* mice (34–37) (**Supplemental Figure 1A**). In fact, diffuse lymphocytic infiltration around bronchi and vessels was observed in the lesions of 20-week-old female MRL/*lpr* mice (**Supplemental Figure S1A**), while mild to moderate lymphocytic infiltration around bronchi and vessels was detected in the pulmonary lesions of 20-week-old female *aly/aly* mice (**Supplemental Figure S1A**). Histopathological analysis showed that pulmonary lesions exist in our pSS model mice as early as they reach their eighth weeks of age (**Figure 1C**). Interstitial fibrosis is known to occur in about half of the pSS patients, accompanied by pulmonary lesions and progressive fibrosing pneumonia that generally leads into severe respiratory failure (38). In the pSS model mice, pulmonary fibrosis is not conspicuous from the 8th until 32nd week of age. On the other hand, the construction of alveolar space and bronchi, thickness of alveolar wall, and any change in the alveolar epithelium were undetectable in the lung tissues of pSS model mice. In addition, no differences between them were observed in body weight at each age and their lifespan. Because fibrosis in this model was hardly observed, the BALT-like lesions may not cause the pulmonary failure showing severe symptoms. It is well known that autoimmune interstitial fibrosis shows more severe pulmonary failure (13, 14, 38). In addition, fibrosis-related genes, such as *Tgfb1*, *Il33*, *Col1a2*, *Col3a1*, and *Col4a1* mRNA, were not upregulated, but rather downregulated, in the lung tissues of pSS model mice, compared with these of control mice (**Supplementary Figure S2**), suggesting that inflammatory response may contribute to the pulmonary lesions in this model through independent pathway of fibrosis. Therefore, we tried to evaluate fibrosis by using bleomycin as an inducing factor of pulmonary fibrosis in our pSS model mice at 8 or 12 weeks of age. Pathological analysis has shown that prominent fibrosis was detectable at 3 weeks after the bleomycin administration in both the control and the pSS model mice at 11 and 15 weeks of age (**Figure 1D**). No differences were observed between these two groups (**Figure 1D**). In addition, no differences were observed in terms of the evaluation performed through the use of Masson’s trichrome staining between these groups at their 11th or 15th weeks of age (**Figure 1E**). The Ashcroft score was used in order to assess fibrosis, and there were no differences identified between them at their 11th or 15th weeks of age (**Figure 1F**). When lung tissue sections were analyzed with Azan staining, fibrosis in both MRL/*lpr* and *aly/aly* mice was hardly observed. Also in these models, fibrosis may be unrelated to the pulmonary pathology (**Supplementary Figure S1B**). These findings demonstrate that pulmonary inflammatory lesions with lymphocytic infiltration were confirmed in our pSS model mice.

### Subpopulations of immune cells in the pulmonary lesions of the model mice

**Figure 2:**
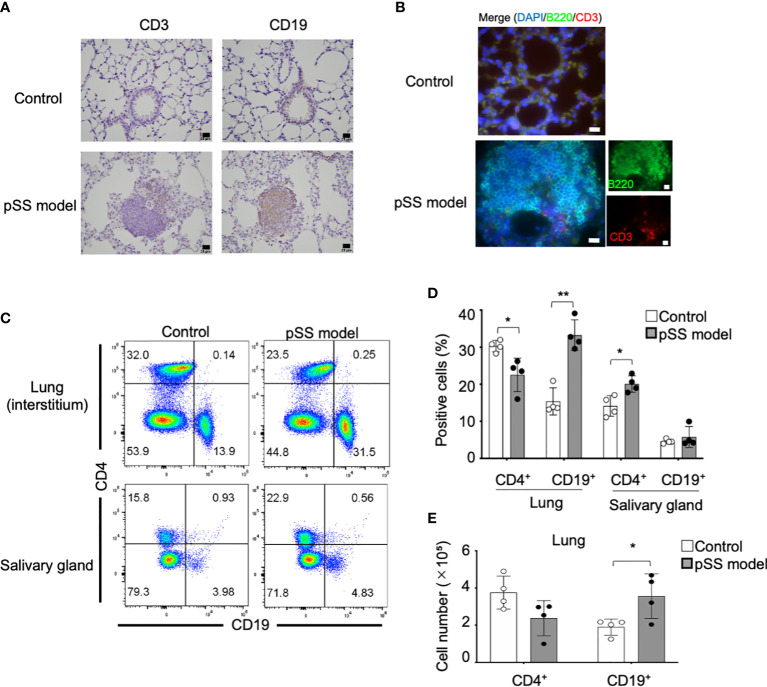
Immunophenotype of the pulmonary lesions identified in pSS model mice. **(A)** CD3^+^ T cells and CD19^+^ B cells in the lung tissues were detected through the immunohistochemical analysis of lung tissue sections obtained from 8-week-old control and pSS model mice. Scale bar: 20 μm. **(B)** CD3^+^ T cells and B220^+^ B cells in the lung tissues were detected through immunofluorescence in sections obtained from 16-week-old control and SS model mice. Scale bar: 20 μm. **(C)** The proportion of CD4^+^ T cells and CD19^+^ B cells in the lung and the salivary gland tissues was evaluated through flow cytometry, by using the isolated lymphocytes (alive) from the tissues of 16-week-old control and SS model mice. Representative results are shown (upper panels: lungs; lower panels: salivary glands). **(D)** The proportions of the CD4^+^ T cells and the CD19^+^ FB cells in the lung and the salivary gland tissues was evaluated through flow cytometry in 16-week-old control mice and SS model mice. Data are presented as mean ± SD of four mice per group. *p < 0.05, **p < 0.01. **(E)** The number of CD4^+^ T cells and CD19^+^ B cells was determined by using the lateral lobes of the lung tissues obtained from 16-week-old control and SS model mice. Data are presented as mean ± SD of four mice per group. *p < 0.05.

T cells among various other immune cells, including B cells, macrophages, dendritic cells, and natural killer cells, are mainly infiltrating in the salivary glands during the early stages of pSS in patients (21). Based on the progression of the inflammatory lesions during the chronic stage of pSS, an increase in the numbers of B cells or plasma cells is also identified in the salivary gland lesions in pSS patients and of mouse models (21, 25). Immunohistochemical analysis has shown that CD19^+^ B cells are mainly infiltrating the pulmonary lesions of the pSS model mice once they reach 8 weeks of age, whereas CD3^+^ T cells were also found (**Figure 2A**). The use of immunofluorescence analysis has also demonstrated that B220^+^ B cells can be prominently observed in the pulmonary lesions of the pSS model mice (**Figure 2B**). In addition, the employed flow cytometry of lymphocytes within the interstitial tissues of the lung has revealed that both the proportion and the cell numbers of CD19^+^ B cells in pSS model mice are significantly increased compared with those of control mice (**Figures 2C–E**). On the other hand, the proportion of CD4^+^ T cells in the salivary gland tissues of the pSS model mice was found to be significantly higher than that of control mice, while there was no difference in terms of the proportion of CD19^+^ B cells between these two groups (**Figures 2C, D**). Our results suggest that B cells may play a key role in the formation of the autoimmune lesions observed in the lungs of the pSS model mice during the early stage of the disease, unlike the inflammatory lesions observed in the salivary glands.

### Enhanced mRNA expression of chemokines and their receptors in the lung tissues of the pSS model mice

**Figure 3:**
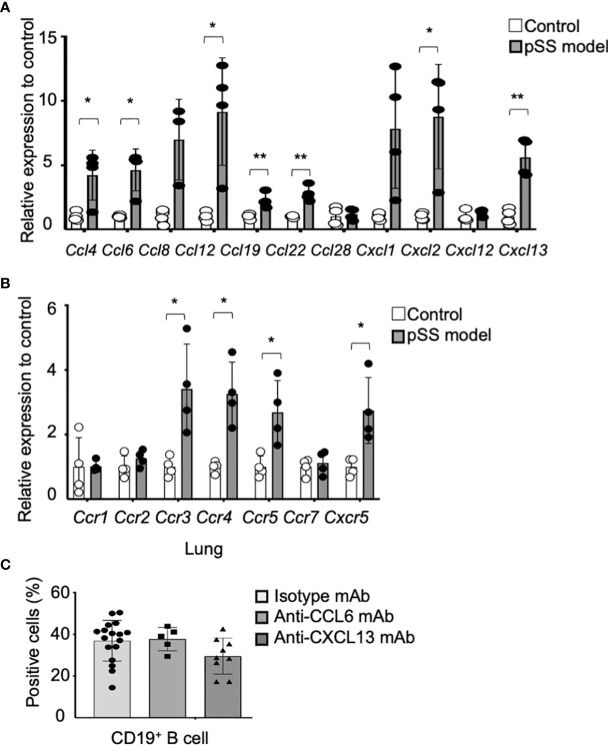
Contribution of chemokines to the formation of pulmonary lesions in pSS model mice. **(A)** The mRNA expression of various chemokine genes was analyzed through qRT-PCR, by using the lung tissues of 8-week-old control and pSS model mice. The relative expression to the respective control mRNA levels is shown, and the data are presented as mean ± SD of three to four mice per group. *p < 0.05, **p < 0.01. **(B)** Chemokine receptor gene mRNA expressions were analyzed through qRT-PCR by using the lung tissues of 8-week-old control and pSS model mice. The relative expression to the respective control mRNA levels is shown, and the data are presented as mean ± SD of three to four mice per group. *p < 0.05. **(C)** The proportion of CD19^+^ B cells infiltrating in the lung tissues of anti-CCL6 or anti-CXCL13 mAb-treated pSS model mice. Data are presented as mean ± SD of 5−17 mice per group. *p < 0.05.

Subsequently, we hypothesized that the peripheral B cells might migrate to the lung tissues through a chemokine network so as to form the inflammatory lesions observed in the pSS model mice. Quantitative reverse transcription–polymerase chain reaction (RT-PCR) analysis has shown that the mRNA expression of various chemokine genes, such as *Ccl4*, *Ccl6*, *Ccl12*, *Ccl19*, *Ccl22*, *Cxcl2*, and *Cxcl13*, in the lung tissues of pSS model mice was significantly higher than that in control mice (**Figure 3A**). Moreover, the mRNA expression of chemokine receptor genes, such as *Ccr3*, *Ccr4*, *Ccr5*, and *Cxcr5*, in the lung tissues of pSS model mice was significantly increased when compared with that of control mice (**Figure 3B**). Among these chemokines, CCL6 has been reported to contribute to the lung inflammation observed in allergic mouse models (39). In addition, CXCL13 is known to play a potent role in the B-cell migration (40, 41). Thus, the anti-CCL6 mAb or the anti-CXCL13 mAb was intraperitoneally administered to pSS model mice from their sixth till the eighth week of their lives. Flow cytometric analysis has revealed that the proportion of B cells in the lung tissues of anti-CCL6 or anti-CXCL13 mAb-treated mice was not significantly altered when compared with that observed in the lung tissues of isotype control mAb-treated mice (**Figure 3C**). Our results suggest that an abnormal chemokine network does not potentially contribute to the B-cell migration in the autoimmune pulmonary lesions in our pSS model mice. Instead, B-cell proliferation or differentiation in the lung may play a key role in the formation of the autoimmune lesions.

### Phenotype of B cells in the pulmonary lesions of the pSS model mice

**Figure 4:**
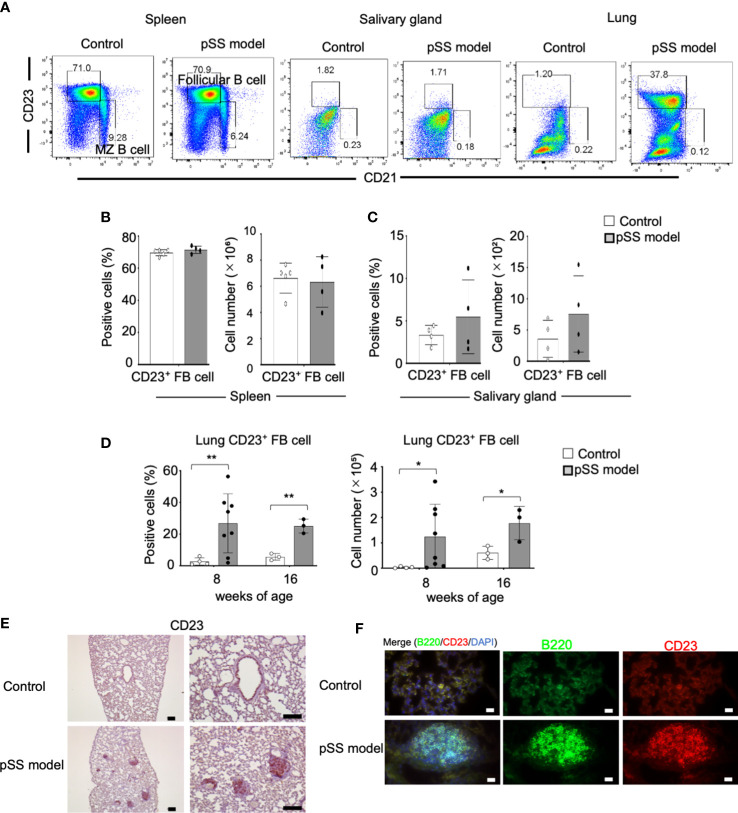
FB cells in pSS model mice. **(A)** F (CD23^+^ CD21^−^) and MZ (CD23^−^ CD21^+^) B cells among the CD19^+^ cells identified in the spleen, the salivary glands, and the lungs of 10-week-old control and pSS model. Representative results are shown. **(B)** Proportions and numbers of follicular B cells in the spleen of 10-week-old control mice and pSS model mice. Data are presented as mean ± SD of four to five mice per group. **(C)** Proportions and numbers of follicular B cells in the salivary gland tissues of 12-week-old control and SS model mice. Data are presented as mean ± SD of four mice per group. **(D)** Population of follicular B cells in the lungs of 8- and 16-week-old control mice and SS model mice. Data are presented as mean ± SD of three to eight mice per group. *p < 0.05, **p < 0.01. **(E)** CD23^+^ cells were detected through immunohistochemical analysis by using lung sections of 8-week-old control and SS model mice. Scale bar: 100 μm. **(F)** B220^+^ B cells and CD23^+^ cells were detected through immunofluorescence analysis by using lung sections obtained from 8-week-old control and pSS model mice. Nuclei were stained with DAPI. Scale bar: 50 μm.

We then examined the B-cell phenotypes in the pulmonary lesions of the pSS model mice. CD23^+^ CD21^−^ follicular (F) and CD23^−^ CD21^+^ marginal zone (MZ) B-cell phenotypes among the mature CD19^+^ B cell subsets were analyzed by using flow cytometry on spleen cells and on lymphocytes obtained from the salivary glands and the lung tissues. The subset representing the CD23^+^ CD21^−^ FB cells in the lungs of the pSS model mice was found to be clearly increased, in contrast to that of control mice (**Figure 4A**). On the other hand, there were no differences among the proportion of the CD23^+^ CD21^−^ B cells from both the spleen and salivary glands between control and pSS model mice (**Figure 4A**). Both the proportions and the numbers of the CD23^+^ CD21^−^ B cells in the spleen and the salivary glands obtained from the pSS model mice were found unaltered when compared with those obtained from control mice (**Figures 4B, C**). Significantly increased proportions and numbers of CD23^+^ CD21^−^ B cells in the lungs of pSS model mice were identified when compared with those of control mice at 8 and 16 weeks of age (**Figure 4D**). Moreover, immunohistochemical and immunofluorescence analyses revealed that CD23^+^ FB cells were distributed in the center of the inflammatory foci of the pulmonary lesions in pSS model mice (**Figures 4E, F**). These findings suggest that CD23^+^ FB cells contribute to the formation of pulmonary lesions in pSS model mice.

### CD23 expression in B cells of the pSS model mice

**Figure 5:**
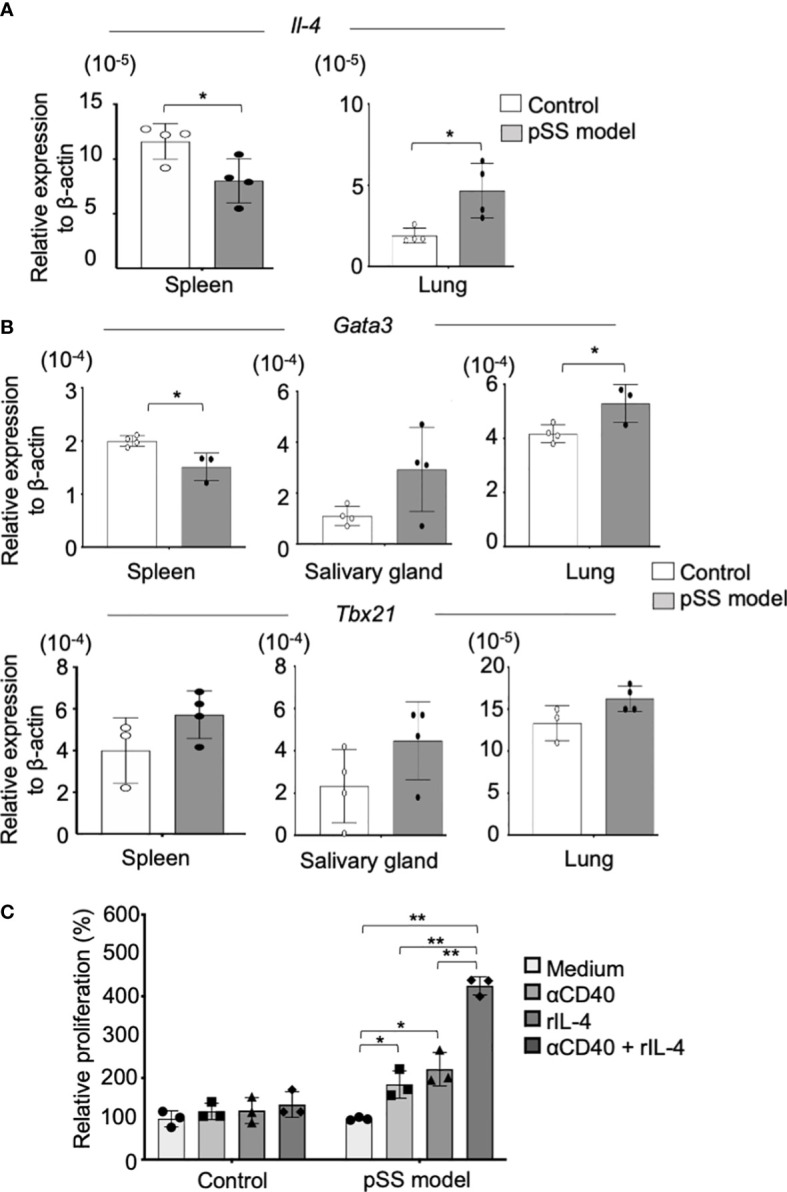
CD23^+^ B-cell differentiation *via* IL-4 in the lungs of the pSS model mice. **(A)** *Il4* mRNA expressions were analyzed through qRT-PCR, by using spleen and lung tissues of 8-week-old control and SS model mice. Data are presented as mean ± SD of four mice per group. *p < 0.05. **(B)** *Gata3* (upper panel) and *Tbx21* (lower panel) mRNA expressions were analyzed through qRT-PCR, by using spleen, salivary gland, and lung tissues of 8-week-old control and SS model mice. Data are presented as mean ± SD of three to four mice per group. *p < 0.05. **(C)** CD19^+^ B cells isolated from the lungs of control and SS model mice were stimulated *in vitro* with an anti-CD40 mAb (5 µg/ml) and recombinant IL-4 (100 ng/ml) for 7 days. The relative cell number of CD23^+^ B cells to the unstimulated cells was evaluated. Data are presented as mean ± SD of triplicates per group. *p < 0.05, **p<0.01.

CD23 upregulation in B cells requires signals of IL-4 and of CD40-CD40L (27). Therefore, we assessed mRNA expression of *Il4* in the lung tissues of pSS model mice. The *Il4* mRNA expression in the lung of pSS model mice was found to be significantly higher than that of control mice, while the expression levels of the spleen of pSS model mice were significantly lower than those in control mice (**Figure 5A**). *Il13* and *Il10* mRNA expression in the lung tissue from pSS model mice was significantly increased compared with that from control mice. IL-13 and IL-10 are well known to be key cytokines for Th2 response and IL-4 (**Supplementary Figure S2**). By contrast, there were no differences in *Ifg-γ* and *Il6* mRNA expression of the lung tissues between control and pSS model mice (**Supplementary Figure S2**). In addition, we evaluated the mRNA expressions of *Tbx21* and *Gata3*, key transcription factors controlling Th1 and Th2, respectively. A significantly decreased mRNA level of *Gata3* in the spleen of pSS model mice was identified when compared with that of control mice (**Figure 5B**, upper panels). The *Gata3* mRNA expression in the lung tissues of pSS model mice was found to be significantly increased when compared with that of control mice, whereas no significant difference was found in the case of the salivary glands (**Figure 5B**, upper panels). By contrast, *Tbx21* mRNA expression levels between the control and the pSS model mice were not different in the spleen, the salivary gland, or the lung tissues examined (**Figure 5B**, lower panels). In order to investigate the direct effect of IL-4 on the CD23 expression in B cells, the latter were isolated from the lung tissues of control and of pSS model mice and were cultured *in vitro* with an anti-CD40 mAb with IL-4, or with a combination of the two, according to a previous report (27). The number of CD23^+^ B cells isolated from pSS model mice was found to be significantly increased after a stimulation with the anti-CD40 mAb or IL-4 (**Figure 5C**). A significantly enhanced number of CD23^+^ B cells was also found after the *in vitro* exposure of pSS model mice-driven B cells to the combination of the anti-CD40 mAb and IL-4 (**Figure 5C**). However, no change was observed in the B cells isolated from the lung tissues of control mice (**Figure 5C**). These results suggest that the Th2 condition in the lungs of the pSS model mice may play an important role in the formation of autoimmune pulmonary lesions including the B-cell infiltration.

### Changes in CD23^+^ FB cells in the pulmonary lesions of the pSS model mice as a result of the anti-CD4 mAb administration

**Figure 6:**
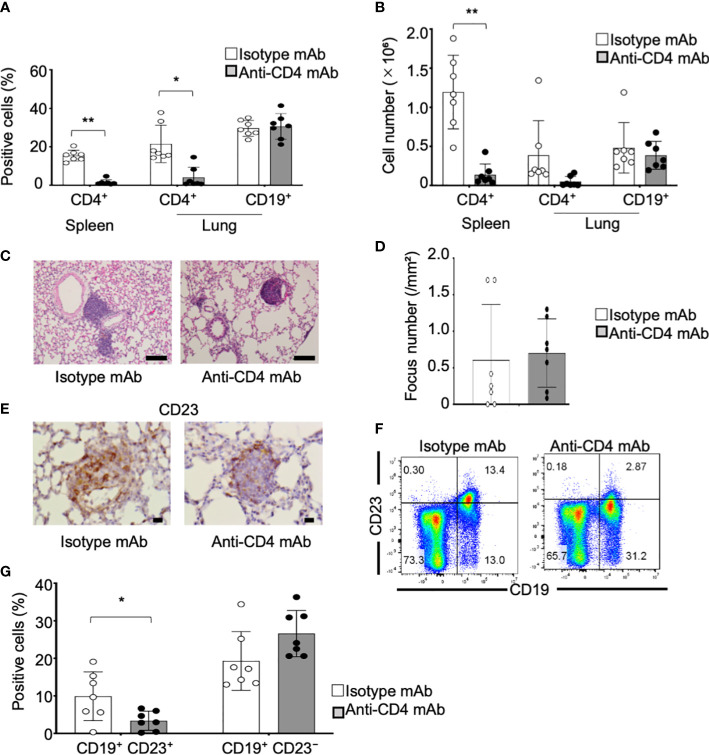
CD23^+^ FB cell differentiation within the lungs of anti-CD4 mAb-treated pSS model mice. **(A)** Anti-CD4 mAb was intraperitoneally administered to pSS model mice between their sixth to eighth week of their lives. We assessed the proportions of CD4^+^ T cells in the spleen and of CD4^+^ T and CD19^+^ B cells in the lungs of isotype control mAb-treated and of anti-CD4 mAb-treated pSS model mice. Data are presented as mean ± SD of seven mice per group. *p < 0.05. **(B)** Number of CD4^+^ T cells in the spleen and of CD4^+^ T and CD19^+^ B cells in the lungs of isotype control mAb- and of anti-CD4 mAb-treated pSS model mice. Data are presented as mean ± SD of seven mice per group. **p < 0.01. **(C)** Pulmonary lesions in anti-CD4 mAb-treated pSS model mice were histologically evaluated. Representative images of HE-stained lung tissues sections of isotype control mAb-treated and anti-CD4 mAb-treated pSS model mice. Scale bar: 100 μm. **(D)** The number of foci in the pulmonary lesions was counted by using HE-stained sections. Data are presented as mean ± SD of seven mice per group. **(E)** CD23^+^ cells in the pulmonary lesions were evaluated immunohistochemically. Representative images are shown for each group. Scale bar: 100 μm. **(F)** CD23^+^ CD19^+^ FB cells and CD23^−^ CD19^+^ B cells were evaluated through flow cytometric analysis by using lung tissues. Representative results are shown for each group. **(G)** The proportions of CD23^+^ CD19^+^ FB cells and of CD23^−^ CD19^+^ B cells were analyzed through flow cytometry. Data are presented as mean ± SD of seven mice per group. *p < 0.05.

CD4^+^ T cells, including follicular T helper (Tfh) and follicular regulatory T (Tfr) cells, play potent roles in differentiation and activation of FB cells in germinal center reaction (42). We have reported that an increased Tfh cells in the periphery contributes to the pathogenesis of autoimmune lesions in the salivary glands of pSS model mice [43]. A significantly increased proportion of the T cells, including Tfh and Tfr cells, was confirmed by flow cytometric analysis using the lung tissues from control and pSS model mice (**Supplementary Figure S3**). Therefore, the anti-CD4 mAb was intraperitoneally administered to pSS model mice from the sixth until eighth week in order to assess whether the CD23^+^ B cells in the pulmonary lesions are controlled by CD4^+^ T cells. The depletion of the CD4^+^ T cells in the spleen and in the lungs was confirmed as a result of the anti-CD4 mAb administration, whereas no changes were found with regard to the CD19^+^ B cells of the lungs in pSS model mice treated with the anti-CD4 mAb (**Figures 6A, B**). The undertaken pathological analysis revealed that the administration of the anti-CD4 mAb has failed to improve the pulmonary inflammation in the pSS model mice (**Figure 6C**). No difference was identified with regard to the focus number/mm^2^ of the pulmonary lesions between the isotype control mAb- and the anti-CD4 mAb-treated mice (**Figure 6D**). On the other hand, the CD23^+^ FB cells in the pulmonary lesions were found to be clearly diminished as a result of the anti-CD4 mAb administration (**Figure 6E**). The proportion of the CD23^+^ FB cells was significantly reduced by the anti-CD4 mAb administration when compared with isotype control mAb administration (**Figures 6F, G**). Our findings suggest that the differentiation of CD23^+^ FB cells in the pulmonary lesions may be controlled by CD4^+^ T cells during the development of the autoimmune pathology observed in the lungs of pSS model mice.

### Preventive effect of the anti-CD4 mAb administration on the pulmonary lesions in pSS model mice

**Figure 7:**
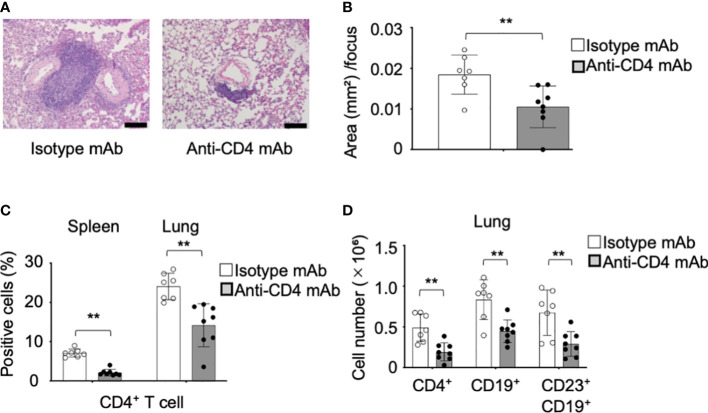
Preventive effect of the anti-CD4 mAb in the pulmonary lesions of pSS model mice. **(A)** Anti-CD4 mAb was intraperitoneally administered to pSS model mice between their fourth to sixth week of their lives. Pulmonary lesions in anti-CD4 mAb-treated pSS model mice were histologically evaluated. Representative images of HE-stained lung tissues sections of isotype control mAb-treated and anti-CD4 mAb-treated pSS model mice. Scale bar: 100 μm. **(B)** The area of foci in the pulmonary lesions was measured by using HE-stained sections. Data are presented as mean ± SD of seven to eight mice per group. **p<0.01. **(C)** We assessed the proportions of CD4^+^ T cells in the spleen and lung of isotype control mAb-treated and of anti-CD4 mAb-treated pSS model mice. Data are presented as mean ± SD of seven to eight mice per group. **p < 0.01. **(D)** Number of CD4^+^ T, CD19^+^ B, and CD19^+^ CD23^+^ FB cells in the lung of isotype control mAb-treated and of anti-CD4 mAb-treated pSS model mice. Data are presented as mean ± SD of seven to eight mice per group. **p<0.01.

To understand the pathogenesis during the onset of the CD23^+^ FB cell-associated pulmonary lesions dependent on CD4^+^ T cells, anti-CD4 mAb was intraperitoneally injected into the pSS model mice from fourth until sixth weeks of their lives. Pathological analysis of the pulmonary lesions showed that the inflammatory foci of anti-CD4 mAb-administered mice were considerably smaller than those of control mice (**Figure 7A**). BALT-like structure was not observed by anti-CD4 mAb administration (**Figure 7A**). The area of the foci of anti-CD4 mAb-treated mice was significantly reduced compared with that of isotype control mAb-treated mice (**Figure 7B**). The depletion of the CD4^+^ T cells in the spleen and in the lungs was confirmed as a result of the anti-CD4 mAb administration (**Figure 7C**). The cell numbers of CD4^+^ T cells, CD19^+^ B cells, and CD23^+^ FB cells of the lung tissues in anti-CD4 mAb-treated mice were significantly decreased compared with those in isotype control mAb-treated mice (**Figure 7D**). The results suggest that the onset of CD23^+^ FB cells-associated BALT-like lesions is dependent of CD4^+^ T-cell activation in the lung of pSS model mice.

## Discussion

In the present study, autoimmune lesions were observed in the lung of a murine model for pSS. A main aspect of the pulmonary lesions of pSS model mice was B cells, unlike the salivary gland lesions in the same model mice. Although the mRNA expression of genes encoding several chemokines and their receptors in the lung tissues was found upregulated in the pSS model mice, the administration of antibodies targeting chemokines responsible for the B-cell migration has failed to inhibit the B-cell infiltration into the lung in pSS model mice. A subset of CD23^+^ FB cells was observed in the pulmonary lesion of pSS model mice. On the other hand, IL-4 in the Th2 condition of lung tissues has enhanced the differentiation of CD23^+^ FB cells in pSS model mice. Moreover, CD4^+^ T cells may play a key role in controlling CD23^+^ FB cells in the pulmonary lesions of pSS model mice. Although the pulmonary lesion with aging has been detectable in the pSS model mice, the precise pathogenesis was clarified in this research.

Since the pSS model was established in our laboratory, a lot of studies have been reported focusing on the salivary and lacrimal glands as the target organs in pSS (24–26, 29–31). By contrast, the pulmonary lesions in this model has been previously described as the secondary lesions followed by the exocrine glands, including salivary and lacrimal glands (43). However, the precise analysis based on pathological and immunological experiments had not been performed. Various clinical and basic studies have been developed in the pathogenesis of SS in the last 30 years. In particular, the clinical feature of extra-glandular lesions in SS, including pulmonary lesion, and the glandular lesion has been clarified. Thus, we investigated the pulmonary lesion of this pSS model to understand the cellular or molecular pathogenesis of SS in comparison with the glandular lesions in the identical model.

The pulmonary lesions of pSS model mice in this study were foci with lymphocytic infiltration, found around the bronchi and the vessels of the lungs. The lesions resembled lymphocytic those of LIP in pSS patients in which small lymphoid aggregations form a germinal center and induce an alveolar septal thickening (15, 16). However, the alveolar septal thickening was not confirmed in our pSS model mice. In addition, BALT has been identified in the lungs of patients with pSS (44–47). It has also been reported that follicular lymphoma, such as the MALT lymphoma, can develop in some pSS patients (48). Interestingly, BALT-like structures were identified in the pulmonary lesions of our pSS model mice. As it has been reported that follicular lymphoma can occasionally occur in some patients with pSS in the form of a MALT lymphoma (48), the formation of BALT in the lungs may be related to the pathogenesis of such a lymphoma. Furthermore, the fibrosis of interstitial pneumonia can also be observed in patients with pSS (49). However, interstitial fibrosis was hardly observed in the pulmonary lesions of our pSS model mice. There was no difference with regard to the interstitial fibrosis induced by bleomycin administration between the control and the pSS model mice. This could be due to the fact that fibrosis may not be closely associated with the pathogenesis of the pulmonary lesions in this model.

Histopathological findings of collagen disease in the lung are so complex for pathologists to diagnose them properly. One of various pathological types of autoimmune pulmonary lesions is similar to the histopathological feature of the pulmonary lesion with BALT-like structure in this pSS model. In order to understand the onset or development of extra-glandular lesions in SS, both glandular and extra-glandular lesions should be analyzed to compare each pathology. The contribution of our study is considerably limited, but the usefulness of this model may contribute to understanding total complex pathogenesis of SS. In addition, it is well known that lymphoid structure is constructed by various kinds of cells and factors. The pulmonary lesion with BALT-like structure consisting of CD23^+^ B cell and so on is also considered to be formed by multiple functions of many cells and factors. For instance, a variety of immune cells, such as follicular T helper cell, follicular regulatory T cell, and follicular dendritic cell, play key roles in the formation lymphoid structure, including germinal center. As a next future study, we have to analyze many immune cells other than CD23^+^ B cells in the pulmonary autoimmune lesion in SS.

Basically, it is difficult to induce fibrosis spontaneously in almost mouse strains, such as gene-manipulated mice. Therefore, a bleomycin-induced fibrosis model is used in many studies. Also in our model, pulmonary fibrosis was hardly observed at even 32nd week of age. In addition, fibrosis-related genes, such as *Tgfb1*, *Il33*, *Col1a2*, *Col3a1*, and *Col4a1* mRNA, were not upregulated in the lung tissues of pSS model mice, compared with these of control mice. On the other hand, inflammatory lesions without fibrosis are known in some patients with SS (17). Thus, analyzing the pulmonary lesion in our model would be useful for understanding a variety of lesions in the pathogenesis of SS.

B cells were found to mainly infiltrate the pulmonary lesions in our pSS model mice. We have previously reported that the numbers of Tfh cells and germinal center B cells in the lymph nodes and the spleen are increased in the pSS model mice (50). In addition, higher levels of autoantibody production, such as that of the anti-SS/A, the anti-SS/B, and the anti-α-fodrin autoantibody, were detectable in the sera of the pSS model mice (50). Although it is still unclear whether such autoantibodies can be produced at the pulmonary lesions of the pSS model mice, the inflammatory foci with a BALT-like structure may contribute to the pathogenesis of pSS as one of its autoimmune responses. As for the other models of SS with pulmonary lesions, the MRL/*lpr* mice have been well known (34). However, a diffuse lymphocytic infiltration (i.e., perivasculitis) can be observed in the pulmonary lesions of MRL/*lpr* mice, unlike the lesions of the pSS model mice used in our study (34). Moreover, mild lymphocytic infiltration can be detectable in *aly/aly* mice, one of the mouse models for SS (35). Many mouse models accompanied by autoimmune pulmonary lesions have been previously reported (51, 52). Although it is difficult to explain the organ specificity for the lung as an autoimmune target, the anatomical structure, the reticuloendothelial system, and the circulatory system of the lung may all be associated with the formation of these autoimmune lesions in pSS.

The mRNA expression of genes encoding several chemokines and their receptors was found to be upregulated in the lung tissues of the pSS model mice when compared with those in control mice. The *in vivo* administration of a mAb for specific chemokine, such as CCL6 and CXCL13, partially inhibited the B-cell migration into the lungs of the pSS model mice, thereby suggesting that a variety of chemokines, but not a single chemokine signal, may contribute to the autoimmune response and the B cell migration observed in the lungs of the pSS model mice. Among the various chemokines, CXCL13 plays a potent role in B-cell migration and in the organization of B cells in the follicles of lymphoid tissues through its receptor, CXCR5 (40, 41, 53). As both the *Cxcl13* and the *Cxcr5* mRNA expressions were found to be upregulated in the lung tissues of pSS model mice when compared with those of control mice, a successful effect of the administered anti-CXCL13 mAb was herein expected. However, only a partial inhibitory effect on B-cell migration into the lungs was observed in the treated pSS model mice. After considering the concentration of the mAb and the administration timing, the CXCL13/CXCR5 axis may be a promising therapeutic target for the pulmonary lesions of pSS. The results in this study suggest that B-cell activation, proliferation, or differentiation in the lung may play an important role in the formation of the autoimmune lesions rather than B-cell migration into the lung tissues.

CD23 is a type II transmembrane glycoprotein containing a Ca^2+^-dependent lectin domain (54–58). CD23 exists in membrane-bound and soluble forms, and increased levels of soluble CD23 are observed in the serum of patients with SS and systemic lupus erythematosus (59). CD23 is a low-affinity receptor for IgE that regulates IgE levels (60). IgE-captured antigens bind to CD23 molecules on B cells and are transported to the B-cell follicles of the spleen. The antigens are transferred from the CD23^+^ FB cells to antigen-presenting cells, such as the dendritic cells or the macrophages, in order to enhance Ab response through the presentation of the antigen to T cells (60). In the present study, the CD23^+^ FB cell population was clearly observed in the lung tissues of pSS model mice. BALT-like structures in the pulmonary lesions may reflect an increase in CD23^+^ B cells in the pSS model mice. Furthermore, the results of our *in vitro* and *in vivo* experiments demonstrate that the differentiation into CD23^+^ FB cells in the pulmonary lesions is enhanced by IL-4 depending on CD4^+^ T cells within the lung tissues of pSS model mice. It has been reported in asthma model mice that the CD23 expression in mature FB cells in their lungs can be upregulated when the Th2-type cytokines are elevated (61). By contrast, Th1 and Th17-type cytokines have been well known to play key roles in the pathogenesis of autoimmune lesion in the salivary and lacrimal glands of both patients with SS and the animal models (3, 28, 62). Thus, the cytokine balance in each target organ may determine the differentiation of B cells related to the formation of the follicular structure in the pulmonary lesions of the herein examined pSS model mice. Moreover, we have a plan to analyze comprehensive gene expression of CD23^+^ B cells in the pulmonary lesion of pSS model mice by single-cell RNAseq as a next future study.

In conclusion, the pulmonary inflammatory lesions with a BALT-like structure were observed in the herein employed murine model of pSS. The CD23^+^ FB cell differentiation that occurs in CD4^+^ T-cell-dependent manner under a Th2-type condition, contributes to the formation of autoimmune lesions in the lungs of the pSS model mice. The results in this study suggest that there is a much greater expansion of CD23^+^ FB cells in the lungs rather than salivary glands in pSS model mice. Our findings shed more light on the pSS pathophysiology and on the identification of potent targets for the treatment of autoimmune diseases.

## Data availability statement

The raw data supporting the conclusions of this article will be made available by the authors, without undue reservation.

## Ethics statement

The animal study was reviewed and approved by Committee on Animal Experiments of Tokushima University (permit number T2021-48). Written informed consent was obtained from the owners for the participation of their animals in this study.

## Author contributions

Conception and design: MS-F and NI. Generate experimental data: MS-F, RA, AU, KO, and TT. Analysis and interpretation: MS-F, RA, AU, KO, TT, RN, SM, and HT. Writing of the manuscript: MS-F and NI. Study supervision: NI. All authors contributed to the article and approved the submitted version.
