# Supplementary material for: CD4+ T-cell-dependent differentiation of CD23+ follicular B cells contributes to the pulmonary pathology in a primary Sjögren’s syndrome mouse model
Source: Front Immunol. 2023 Jul 5;14:1217492. doi: 10.3389/fimmu.2023.1217492 (PMC10354287; doi:10.3389/fimmu.2023.1217492)
Supplement: Supplementary file 1 [file DataSheet_1.docx]

Supplementary Material

CD4^+^ T cell-dependent differentiation of CD23^+^ follicular B cells contributes to the pulmonary pathology in a primary Sjögren’s syndrome mouse model

Mami Sato-Fukuba ^1,2^, Rieko Arakaki ^1^, Aya Ushio ^1^, Kunihiro Otsuka ^1^, Ruka Nagao ^1^, Shigefumi Matsuzawa ^1^, Hiroaki Tawara ^1^, Takaaki Tsunematsu ^1^, Naozumi Ishimaru ^1*^

^1^Department of Oral Molecular Pathology, Tokushima University Graduate School of Biomedical Sciences, Tokushima, Japan

^2^Department of Oral Medicine, Tokushima University Hospital, Tokushima, Japan*****

**Correspondence:** Naozumi Ishimaru: isimaru.n@tokushima-u.ac.jp

## Supplementary Figures


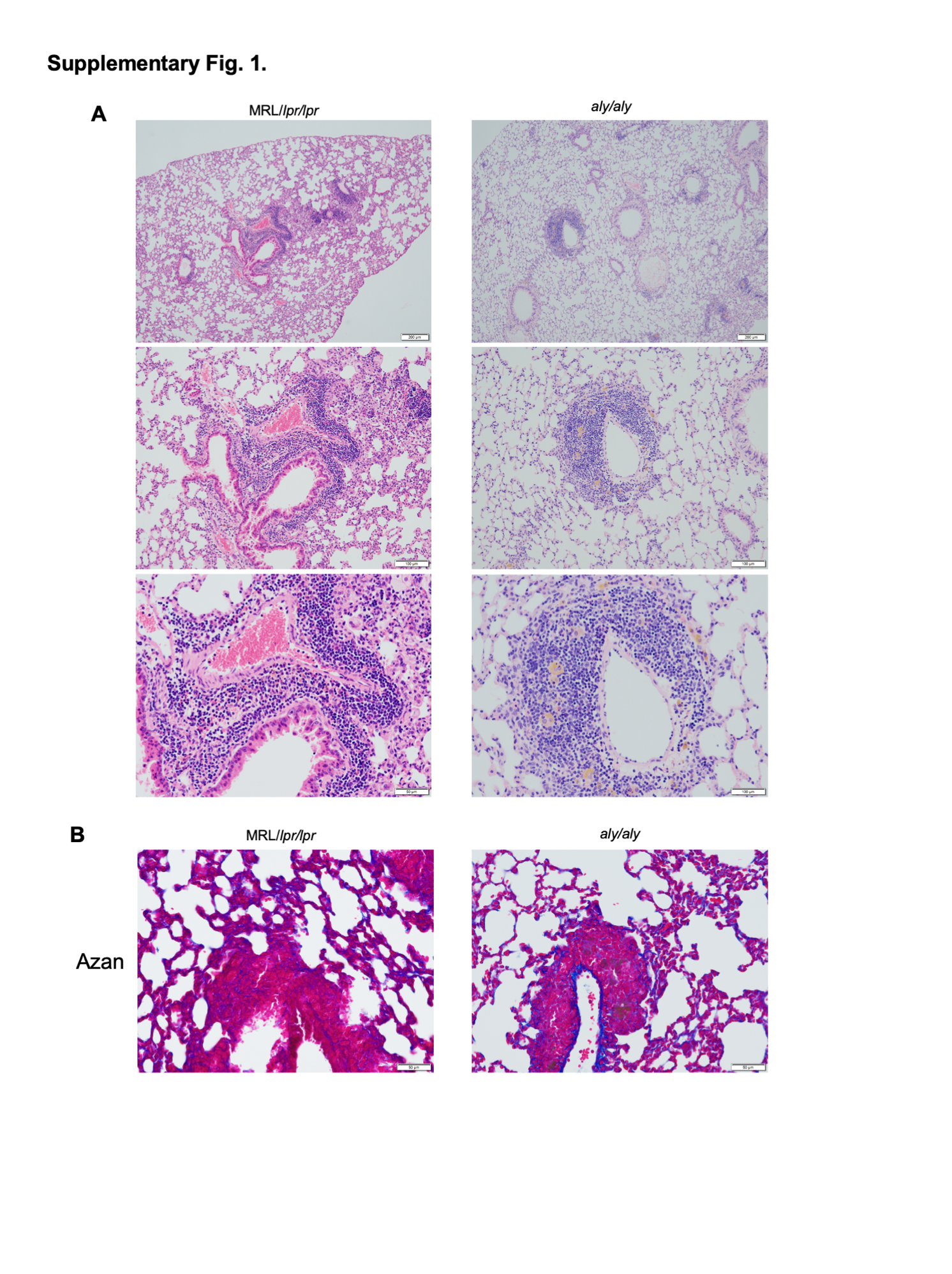


**Supplementary Figure 1.** **A**. The pulmonary lesions identified in female MRL/*lpr* and *aly/aly* mice, among those of other SS model mice, were histopathologically assessed. Representative images of HE-stained sections obtained from 20-week-old the aforementioned model mice. Scale bar: 200 µm (upper panels), 100 µm (middle panels), and 50 µm (lower panels). **B.** Connective tissue was detected by Azan staining using lung tissues of MRL/*lpr* and *aly/aly* mice. Scale bar: 50 µm.

**
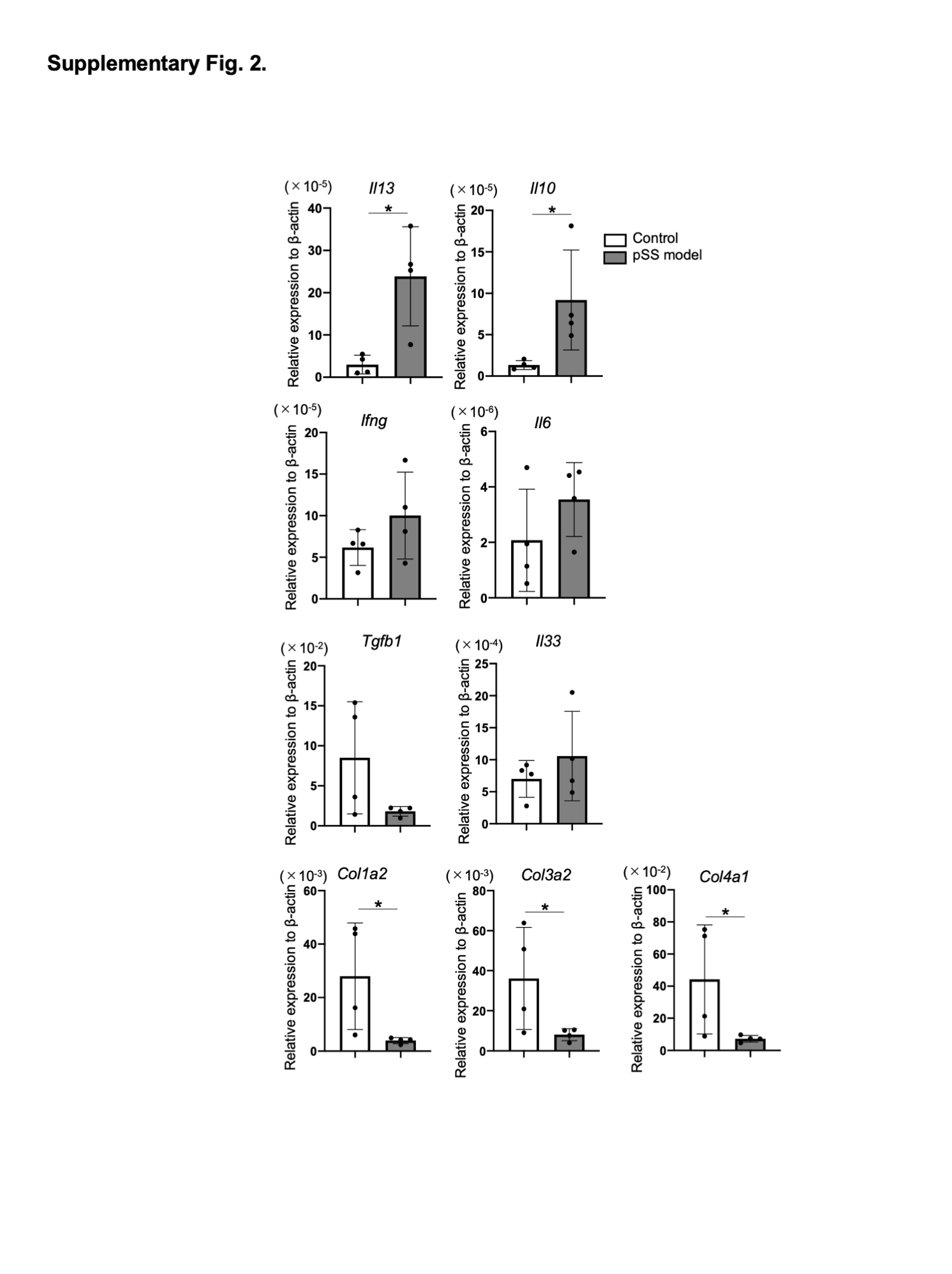
**

**Supplementary Figure 2.** mRNA expressions of inflammatory cytokines, including *Il13*, *Il10*, *Ifng*, *Il6*, *Tgfb1*, *Il33*, *Col1a2*, *Col3a2*, and *Col4a1*, were analyzed by qRT-PCR using lung tissues from control and pSS model mice. Data are presented as mean ± SD of 3-4 mice per group. **p* < 0.05.

**
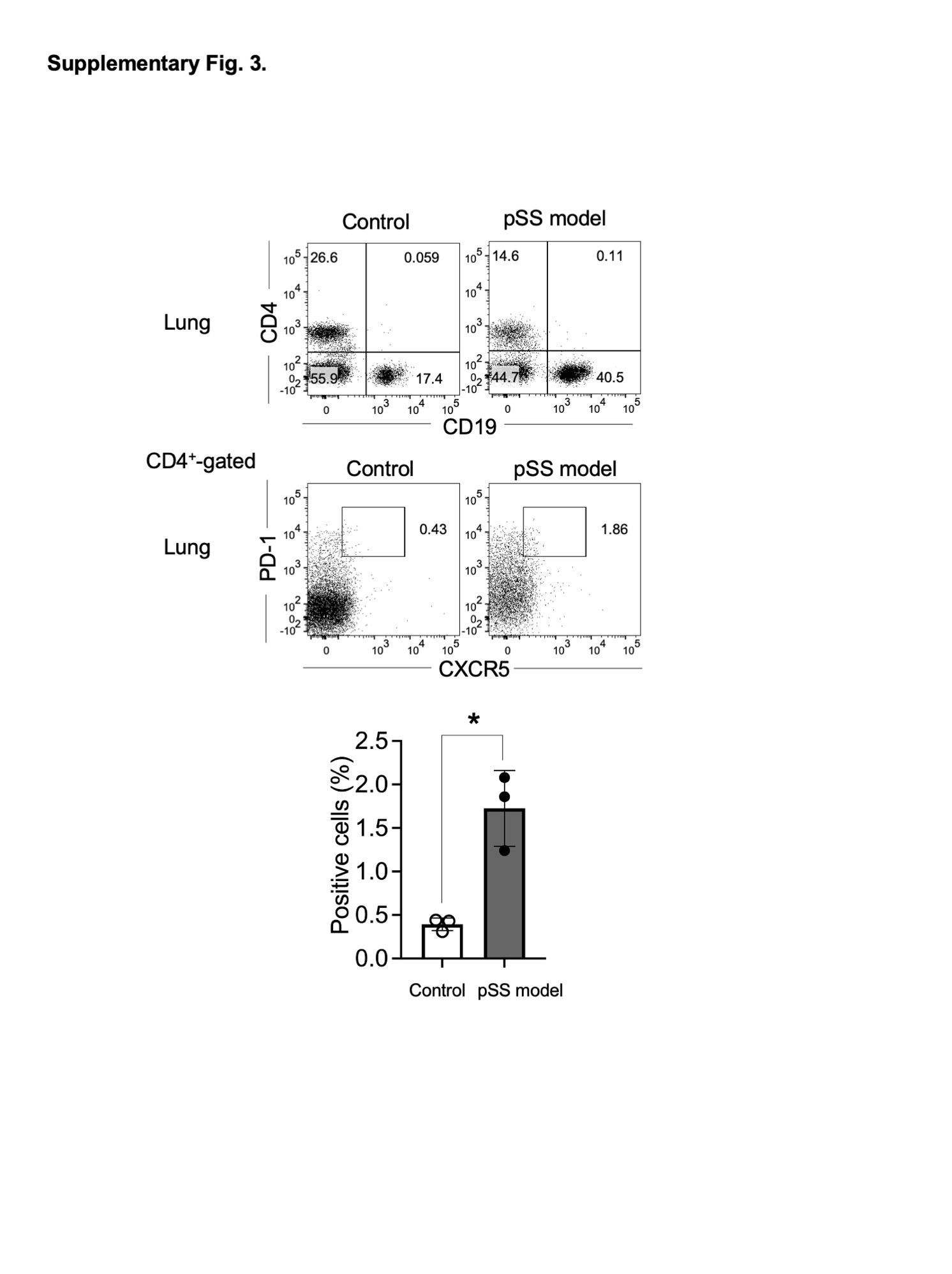
**

**Supplementary Figure 3.** CD4^+^PD-1^+^CXCR5^+^ T cells, including Tfh and Tfr cells, in the lung tissues from control and pSS model mice were detected by flow cytometric analysis. Data are presented as mean ± SD of 3 mice per group. *p < 0.05.
